# Organophosphorus Chemistry 2021

**DOI:** 10.3390/molecules28010394

**Published:** 2023-01-03

**Authors:** György Keglevich

**Affiliations:** Department of Organic Chemistry and Technology, Budapest University of Technology and Economics, 1521 Budapest, Hungary; gkeglevich@mail.bme.hu

Forty years have passed since a real “phosphabenzene” (a phosphinine) was prepared for the first time applying steric protection methodology with the sterically demanding 2,4,6-tri-tertiary-butylphenyl (“supermesityl”) group. It was generally believed that molecules, such as those with heavier main-group elements, had never existed as stable compounds, but various kinds of “unusual” phosphorus compounds could be isolated and characterized by utilizing the stabilizing effect of the “supermesityl” group. Several sterically protected, low-coordinate organophosphorus compounds with P=P, P=C, and C≡P bonds are described in this study by the founder of this topic, Yoshifuji [1]. Molecules, such as diphosphenes, phosphaalkenes, 1- phosphaallenes, 1,3-diphosphaallenes, 3,4-diphosphinidene-cyclobutenes, and phosphaalkynes, were stabilized with the bulky “supermesityl” group. The synthesis, structures, and physical and chemical properties of these molecules were surveyed together with a few successful applications in catalytic organic reactions. There is a high interest in this class of compounds and synthetic applications in basic research.

A synthetic protocol for the preparation of novel phosphinic dipeptides was presented by Georgiadis and co-workers [2]. These compounds serve as valuable building blocks for developing highly potent phosphinopeptidic inhibitors of medicinally relevant Zn-metalloproteases and aspartyl proteases. The proposed method was based on the tandem esterification of α-aminophosphinic and acrylic acids under silylating conditions to subsequently participate in a phospha-Michael addition. The scope of the efficient transformation was extended to a diverse set of acrylic acids and (R)-αaminophosphinic acids. In most cases reported herein, the isolation of biologically relevant (R,S)-diastereoisomers was possible by simple crystallization from the crude mixtures, enabling a practical and simple method. Based on the new results, it may be expected that the protocol introduced will facilitate the discovery of pharmacologically useful bioactive phosphinic peptides.

A review survey showed that hydrolysis is the most frequently used method for preparing P-acids [3]. The products, various P-acids, have a significant role in drugs, herbicides, and flame retardants. The process may be catalyzed by acids and bases. Comparing the two methods, it can be said that the alkaline accomplishment takes place faster and is less corrosive, but it is realized in two steps. The course of the reactions may be influenced by various factors, such as temperature, solvent, pH, and the substituents attached to the phosphorus atom. The steric hindrance decreases the reaction rate, while the electron-withdrawing effects in the C- or OC-substituents attached to the P atom increase the reactivity. Although the hydrolyses of P-esters have not yet been explored fully, and in general, the reaction conditions have not been optimized, this article tries to demonstrate how to accomplish hydrolyses efficiently. In addition to hydrolysis under conventional conditions, MW-assisted variations were also demonstrated, with environmentally friendly and even more efficient accomplishments. 

Although the chemical synthesis of dendrimers is more than two decades old, the most significant reason hampering the broader use of dendrimers in biomedicine is usually the difficult, time-consuming multistep preparation, especially for the high-generation structures. Consequently, the development of methodologies offering more attractive access to these macromolecular materials is especially needed. Salamonczyk introduced an easy and highly efficient method for synthesizing new polyanionic dendrimers as potential broadspectrum antiviral drugs [4]. The mild conditions of the coupling and deprotection reactions provided highly pure and water-soluble macromolecular materials in good overall yields. The important advantage of the presented strategy is direct access to the polyanionic material at each generation of the prepared dendrimer. This approach seems to be a general methodology that enables the transformation of practically any macromolecular compound terminated with hydroxy functions into its polyanionic derivative. Moreover, it offers the possibility to make discrete modifications layer-by-layer (i.e., P=O, P=S, and/or carbon branching) within the same dendrimer skeleton, key for a structure–activity relationship study.

Nycz et al. summarized the available results on molecules containing the >P(=O)-P(=O)< fragment, which notably resembles the structure of the >P(=O)-O-P(=O)< moiety [5]. Both structural motifs are essential building blocks for many important molecules found in nature and the field of medicinal chemistry. The review covers the strategies related to synthesizing hypodiphosphoric acid (former name hypophosphoric acid), its ester form, and diphosphine dioxides. Last but not least, a few properties and applications of the compounds with the outlined structures are presented.

The next article discusses the Pudovik reaction between α-oxophosphonates (ZC(O)P(O)(OEt)_2_, Z = Me or Ph) and Y_2_P(O)H reagents (Y = EtO, MeO, and Ph) that may lead to the corresponding adducts (Y_2_P(O)C(OH)ZP(O)(OEt)_2_), a kind of dronic acid derivatives, and/or their rearranged versions [6]. The outcome mostly depended on the Z substituent, the quantity of the dialkylamine catalyst, and, to a lesser extent, the nature of other substituents, as well as the temperature and the solvent. In a few cases, time also influenced the course of the reaction. In cases where Z = Me, the adducts were the primary products, but with suitable modifications, the reactions could be tuned to yield the rearranged derivatives. At the same, in cases where Y = Ph, the corresponding adducts were only intermediates that were converted spontaneously to their rearranged versions. This phenomenon was explained by electronic factors. In reaction with dimethyl phosphite and diphenylphosphine oxide, the rearranged species comprised two isomers. 

Dronic acid derivatives, important drugs against bone diseases, may be synthesized from the corresponding substituted acetic acid either by reaction with phosphorus trichloride in methanesulfonic acid as the solvent or by also using phosphorous acid as the P-reactant if sulfolane is applied as the medium [7]. The possible mechanistic pathways leading to fenidronic acid and benzidronic acid were computed considering the reaction of benzoic acid or phenylacetic acid and derivatives with PCl_3_ in MSA or with PCl_3_/P(OH)_3_ in sulfolane. It was found that the corresponding acyl chlorides should be considered as the starting compounds, and PCl_3_ or its condensed derivative with MSA or H_3_PO_4_ (Cl_2_P-O-SO_2_Me or Cl_2_P-O-P(OH)_2_, respectively) may be the nucleophile. The multistep formation of the bis adducts was, in most cases, endothermic, and the exothermic final hydrolyses could be the driving force for the series of reactions. In the case of benzidronic acid, the activation Gibbs free energies were lower for all steps than for fenidronic acid. It is noteworthy that in the case of using PCl_3_/P(OH)_3_ in sulfolane, the formation of the adducts was exothermic. It turned out that the setup with PCl_3_/P(OH)_3_ in sulfolane involving (HO)_2_P-O-PCl_2_ as the nucleophile is the energetically preferable option. Hence, in the synthesis of dronic acid derivatives, this approach is recommended.
